# Quality of care and health status in Ukraine

**DOI:** 10.1186/1472-6963-14-446

**Published:** 2014-09-30

**Authors:** John W Peabody, Jeff Luck, Lisa DeMaria, Rekha Menon

**Affiliations:** QURE Healthcare, 1000 Fourth Street, Ste 300, San Rafael, CA 94901 USA; University of California, 50 Beale Street, 12th Floor, San Francisco, CA 94105 USA; College of Public Health and Human Sciences, Oregon State University, 401 Waldo Hall, Corvallis, OR USA; Tanzania, Uganda and Burundi, Human Development Sector Unit, Africa Region, The World Bank, Room 410, 50 Mirambo Street, P. O. Box 2054, Dar Es Salaam, Tanzania

**Keywords:** Quality of care, Health care delivery, Non-communicable diseases, Health policy, Ukraine, Eastern Europe

## Abstract

**Background:**

We conducted a national level assessment of the quality of clinical care practice in the Ukrainian healthcare system for two important causes of death and chronic disease conditions. We tested two hypotheses: a) quality of care is predicted by physician and facility characteristics and b) health status is predicted by quality of care.

**Methods:**

During 2009–2010 in Ukraine, we collected nationally-representative data from clinical facilities, physicians, Clinical Performance and Value (CPV®) vignettes, patient surveys from the facilities, and from the general population. Each physician completed a written CPV® vignette—a simulated case scenario of a typical patient visit—for each of two clinical cases, congestive heart failure (CHF) and chronic obstructive pulmonary disease (COPD). CPV® vignette scores, calculated as a percentage of all care criteria completed by the physician, were used as the measure of clinical quality of care. Self-reported health measures were collected from exit and household survey respondents. Regression models were developed to test the two study hypotheses.

**Results:**

136 hospitals and 125 polyclinics were surveyed; 1,044 physicians were interviewed and completed CPV® vignettes. On average physicians scored 47.4% on the vignettes. Younger, female physicians provide a higher quality of care—as well as those that have had recent continuing medical education (CME) in chronic disease or health behaviors. Higher quality was associated with better health outcomes.

**Conclusions:**

As low- and middle-income countries around the world are challenged by non-communicable diseases, higher quality of care provided to these populations may result in better outcomes, such as improved health status and life expectancy, and overcome regional shortfalls. Policy efforts that serially evaluate quality may improve chronic disease care.

## Background

In most countries today, whether rich or poor, a growing burden of non-communicable diseases (NCDs) is a significant policy issue. The high prevalence and resulting burden of chronic diseases pose challenges to health care systems to adequately diagnose and treat individual patients.

Over the past 20 years, Ukraine has been undergoing a health crisis [1]. Life expectancy is increasing gradually following a sharp drop after independence [2], but remains below most other European countries, at 68 years compared to 79 for Europe as a whole; worse still, 13% of these years (7.7 years in total) are spent in poor health [3]. NCDs already account for a staggering 82% of all deaths in the country and are especially preventable or treatable [3]. The most common NCDs—coronary heart disease (CHD), congestive heart failure (CHF), and chronic obstructive pulmonary disease (COPD), along with hypertension and diabetes—are major contributors to the declining life expectancy in Ukraine and other post-Soviet countries [4].

The impact of NCDs on health outcomes and perceptions of inadequate care in many low and middle-income countries (LMICs) have heightened interest in the quality of clinical care, particularly for diagnosing and treating cardiovascular (CVD) [5] and chronic respiratory disease (CRD). Better diagnosis and treatment, particularly of associated risk factors, has led to improved CVD and, to a lesser extent CRD, outcomes in more developed economies [5].

Thus there is an underlying urgency to understand how the quality of clinical care can be measured at a population level in LMICs [6–8]. Recent research from developed and developing countries shows that quality can be measured at the community level. Extending this work to a national level assessment of the quality of care for NCDs, might make it possible to relate quality of care to health status at the patient or even the community level [9].

We conducted a national study of the quality of care in Ukraine, a middle-income country where, despite some incremental reforms in the past two decades [10], there is a pressing need for improved health system effectiveness [11]. Most care is delivered by employed physicians in government-operated facilities, including polyclinics and hospital outpatient clinics. Although the tax-financed system is nominally free at the point of care, it faces challenges of underinvestment and widespread informal payments [12].

We used two practical, affordable measurement tools to evaluate the quality of care for, and health impacts of, NCDs on a population scale. The first is measurement using simulated cases. Physicians and other providers care for patients as they would an actual patient, to demonstrate and benchmark how their care compares to evidence-based practice [13, 14]. Clinical Performance and Value (CPV®) vignettes, simulations that have been used widely in different countries and practice settings, are affordable [15, 16] and have been validated against actual clinical practice [13, 14]. Moreover, improvements in CPV scores have been experimentally linked to better patient outcomes. The second advance is the increasing reliance on self-reported health status measures as a measure of health status in population studies [17–19]. A series of studies have established that General Self-Reported Health (GSRH) status is a robust predictor of overall health status and a predictor of future health expenditures, and is related to clinical practice [20, 21].

We conducted a national level assessment of the quality of care in the Ukraine healthcare system to for COPD and CHF, two of the most important causes of NCD burden and mortality. We tested two hypotheses: 1) quality of care for NCDs can be predicted by physician and facility characteristics that are amenable to policy interventions and 2) the quality of care as measured by CPVs predicts health status of individuals using the facilities and the population as a whole.

## Methods

Data for this study were derived from a coordinated series of surveys conducted in Ukraine during 2009 and 2010. We collected data from clinical facilities, physicians, CPV® vignettes for COPD and CHF, patient surveys at the facilities, and a household survey of the general population. The 2009 household survey collected data from over 3,000 adults in 1,408 households. In 2010, data were collected at over 250 health care facilities that served residents of geographic areas sampled in the household survey.

### Sampling and data collection

#### Household survey

The household survey was the foundation for a sampling strategy designed to link population and patient health data with structural and quality measures of the health care delivery system. The household survey collected data in 8 of 27 *oblasts*, (equivalent of a state or province) including the capital city of Kiev and the Autonomous Republic of Crimea. These oblasts were selected to be representative of 4 major regions of Ukraine—West, North/Center, East, and South—and are home to approximately 18 M of the country’s 46 million residents. In each oblast (except urban Kiev), 22 enumeration areas (EAs) were randomly selected, equally divided between rural and urban areas. Neighborhoods were then randomly selected within each EA, and households randomly selected within neighborhoods. All adults in each selected household were interviewed [1].

#### Facility survey and structural quality of care measures

Next we sampled facilities that served residents of the household survey EAs. Each polyclinic serving one or more EAs was identified and designated as either urban serving or rural-serving based on the EAs it served. Polyclinics were then randomly selected to cover as many EAs as possible (given budget constraints) and achieve a distribution of polyclinics comparable to the national urban:rural population ratio. The rayon or city hospital to which each sampled polyclinic referred patients was then selected; its urban or rural designation matched that of the referring polyclinic(s). The oblast hospital in each oblast was also included and was designated as urban-serving.

A total of 136 hospitals and 125 polyclinics were surveyed (Table 1). On average, facilities were more than 20 years old. The facilities served large populations with substantial inpatient (bed) capacity and had long operating hours, such as polyclinics that were open an average of 58.7 and 72.7 hours a week for emergency care. Each facility was staffed by a large number of providers (averaging 180, 78 and 43 doctors at oblast hospitals, rayon hospitals and polyclinics, respectively) seeing many outpatients a month. Polyclinics averaged 20,013 outpatient visits a month, while oblast and rayon hospitals averaged 14,631 and 13,259, respectively. A large fraction of patient volume was for older patients, presumably patients more likely to have chronic or even multiple conditions.

**Table 1:** **Facility characteristics, by facility type**

	All facility types	Oblast hospital	Rayon/Town hospital	Polyclinic
	N = 261	N = 8	N = 128	N = 125
***General Facility Information***				
**Age of facility**	25.5	51.3	28	21.1
	[29.4]	[73.02]	[32.1]	[19.4]
**Population served**	129,589.7	2,418,672	132,326.3	52,567.2
	[431,184.6]	[1,197,941]	[397,339.5]	[83,040.9]
**Number of licensed beds/facility (hospitals)**	332.6	894	296.7	
	[252.9]	[326.3]	[201.2]	
**Number of beds in use/facility (hospitals)**	302.1	892.4	263.1	
	[239.6]	[328.9]	[173.7]	
**Number of listed committees or bodies in place**	3.1	4.3	3.5	2.7
	[1.7]	[1.5]	[1.7]	[1.6]
**Clinical Hours**				
**Total hours open (Monday-Sunday)**	100.1	152.5	138.5	58.7
	[53.9]	[43.9]	[49.3]	[9.8]
**Total hours for emergencies**	132.8	168	165.8	72.7
	[51.1]	[0.0]	[12.01]	[38.8]
	**All facility types**	**Oblast hospital**	**Rayon/Town hospital**	**Polyclinic**
***Patient load***				
**Total number of doctors**	64.5	179.6	78.02	42.8
	[64.4]	[122.4]	[69.5]	[36.1]
**Number of nurses, other clinical staff (e.g. medical technicians, pharmacists)**	195.5	681.9	262.2	90.6
	[202.4]	[446.2]	[186.9]	[75.3]
**Number of OP visits/month**	17,587.1	13,259.2	14,631.2	20,013.4
	[16,794.5]	[8,440.3]	[15,978.1]	[17,339.5]
**Number of inpatient admissions/month (hospitals only)**	1,058.8	4,624.3	857.5	
	[1,268.95]	[3,241.5]	[651.92]	
**% adult patients (>18)**	73.8	61.5	71.9	76.4
	[30.1]	[36.9]	[31.1]	[28.7]
**% of adult patients >65**	21.2	16.3	19.5	23.2
	[15.4]	[9.6]	[13.04]	[17.6]
	**All facility types**	**Oblast hospital**	**Rayon/Town hospital**	**Polyclinic**
***Facility Equipment/Supplies***				
**% of equipment in good working condition (out of 16 items)**	46.0	80	55.7	34.7
	[18.2]	[10.1]	[15.3]	[12.9]
**% of tests performed (out of 15 items)**	76.9	92.2	79.7	73.0
	[19.1]	[9.9]	[15.3]	[21.97]
**% of supplies available (out of 12 items)**	74.8	85.4	79.2	69.6
	[18.1]	[19.3]	[15.8]	[18.8]
Annual Expenses/facility*	$2,220,828	$6,411,543	$2,208,281	$724,702
	[49,600,000]	[21,400,000]	[65,100,000]	[6,789,152]

*Average 2009 exchange rate of 7.97hryvna = USD$1. http://www.tradingeconomics.com/ukraine/currency.

Data on structural quality included human, material and financial resources as well as services and productivity data. We constructed three indices—based on lists used successfully in facility surveys in other countries and reviewed for appropriateness in the Ukrainian context—to quantify the availability of materials needed to provide basic outpatient and inpatient health care: the availability of medical equipment in good working condition; laboratory tests available at the facility; and the availability of basic clinical supplies (Table 1). Each possible equipment item, laboratory test, or supply item was recorded as available or not, and indices present the proportion of items available.

In general, hospitals had higher values of the structural quality indices than did polyclinics. However, there was significant variation in the working order of equipment across facilities (on average, 56% at hospitals and 35% at polyclinics), the percentage of tests performed and the adequacy of supplies across and within facilities of the same type (See Table 1).

#### Physician survey and clinical quality of care measures

We then randomly sampled eligible physicians within each sampled facility. Eligible physicians were those with training in primary care (internal medicine, family medicine, general practice, or social medicine) or relevant specialties (pulmonology/TB for the COPD case or cardiology for the CHF case). At each site, eligible physicians were rostered and four were randomly selected and invited to participate. Of physicians who were approached, 61% agreed to complete the physician survey and CPV® vignettes. Of physicians who refused, 93% cited questionnaire length or lack of time as reasons; 6% cited reluctance to provide confidential information.

The physician survey gathered information about training, current practice, patient load and socio-demographic and economic indicators. We surveyed a total of 1,044 physicians (Table 2). Three quarters were women, with an average age of 47 with 22 years of experience, roughly equally split between Ukrainian speaking and Russian speaking; 1 in 6 to 7 worked at another facility. There were more primary care physicians and numbers of patients seen per month in polyclinics than hospitals, although roughly all facilities had the same proportion of patients over 40.

**Table 2:** **Physician characteristics and quality of care**

Characteristics	All facility types	All hospitals	Oblast hospital	Rayon/Town hospital	Polyclinic
			(N = 34)	(N = 509)	(N = 500)
**Social Demographics**					
**Gender (male) %**	24.7	35.2	35.3	28.3	20.4
**Age**	47.3	47.5	45.4	47.6	47.2
	[11.1]	[11.1]	[13.2]	[10.9]	[11.1]
**Language spoken at home (%):**					
Ukrainian	43.4	42.9	45.2	42.8	44
Russian	41.7	42.7	32.3	43.4	40.5
Both	13	13.1	22.6	12.5	13
Other	2	1.3	0	1.4	2.6
**Medical Training**					
**Years practicing as physician**	22.2	22.5	20.7	22.6	21.9
	[10.7]	10.7	[13]	[10.5]	[10.9]
**Years practicing at the facility**	17.6	17.7	14.1	17.9	17.4
	[10.5]	10.4	[10.1]	[10.4]	[10.7]
**Patient Load**					
**General Practitioners (primary care) (%)**	25.9	11.2	5.9	11.6	41.8
**Patients ≥ 40 years old (%)**	58	57.2	54.8	59.5	56.6
**Hours/week at the facility**	42.6	43.8	39.8	44.1	41.4
	[11]	11.3	[9.6]	[11.4]	[10.5]
**Inpatients per month (number)**	28.7	53.1	27.4	40.7	16.9
	[54]	88.2	[23.9]	[65.4]	[37.9]
**Outpatients/month (number)**	274.3	107.7	176.6	132.6	423.7
	[276.4]	137.8	[301]	[214]	[252]
**Minutes per OP visit**	36.6	51.0	52.7	51.3	27.2
	[53.7]	[72.57]	[74.7]	[72.6]	[33.5]
**Work in other facilities (%)**	13.5	13	18.2	12.8	13.9
**Hrs/week at other facilities**	20.1	21.4	11.8	22.2	18.7
	[16.1]	16.3	[6.1]	[16.6]	[16]
**Case Type**					
**All COPD**	41.5 ± 12.7	41.9 ± 12.5	41.7 ± 10.7	41.9 ± 12.6	41.1 ± 13.1
**All CHF**	53.2 ± 13.1	53.2 ± 13.1	57.3 ± 11.6	52.9 ± 13.2	53.2 ± 13.0
**All Cases**	47.4 ± 11.4	47.5 ± 11.1	49.5 ± 8.7	47.4 ± 11.3	47.2 ± 11.6
**By Domain (combined COPD + CHF)**					
**History**	59.9 ± 11.0	59.8 ± 10.9	61.8 ± 8.7	59.6 ± 11.0	60.0 ± 11.1
**Physical exam**	54.4 ± 23.8	55.6 ± 23.0	56.9 ± 24.8	55.5 ± 22.9	53.1 ± 24.5
**Laboratory tests**	77.5 ± 24.2	78.0 ± 23.9	81.4 ± 18.8	77.7 ± 24.2	77.0 ± 24.8
**Diagnosis**	56.0 ± 14.6	56.2 ± 15.3	58.8 ± 14.1	56.0 ± 15.3	55.8 ± 13.8
**Treatment/Management**	40.9 ± 12.9	40.8 ± 12.7	42.1 ± 12.1	40.7 ± 12.8	41.0 ± 13.2

Each physician also completed 2 Clinical Performance and Value® (CPV®) vignettes, which measured quality of clinical care. A CPV® vignette is a written case simulation of a typical visit from a patient with a given condition. Physicians are asked to take a history, describe the physical examination, order tests, make a diagnosis, and prescribe treatment as they would in an actual practice setting. CPV® vignettes have been validated against actual physician practice in prospective randomized trials in developed country settings [13, 14] and produce comparable results in developing countries [16]. They are capable of detecting changes in physician behavior and quality of care across time [22]. Most recently improvements in CPV® vignette scores have been experimentally linked to improved health status, strongly linking health outcomes to actual clinical practice in LMICs.

For this study, CPV® vignettes were developed for COPD and CHF. These two NCDs are strongly associated with an aging population and carry a high burden of disease. CHF, the end stage of many cardiac conditions, accounts for 38.4% of all deaths in Ukraine [4]; COPD is the third leading cause of death and accounts for 2.9% of all deaths, although this is almost certainly under-reported [4]. A simple and a complex CPV® vignette were developed for each disease.

Physicians were randomly assigned one complex and one simple CPV® vignette case across the two diseases. Completed CPV® vignettes were translated from Ukrainian or Russian into English and then scored by trained physician abstractors, using a predefined set of criteria of care for each CPV® vignette. Physician abstractors have specialty training appropriate for the CPV® vignette cases, and a sample of the CPV® vignettes were over-read by the lead author to ensure accuracy. Scoring criteria were based on US and international standards of care for the two diseases [23, 24] and reviewed by an expert panel of Ukrainian physicians. For each CPV® vignette, scores were calculated as the percentage of items (40–55 criteria for each case) “done” correctly. A total percent score was calculated along with scores for each of five major domains: history taking, physical exam, laboratory testing and imaging, diagnosis, and treatment and management. Historically, the highest CPV® scores range from 65-80%, for very good to excellent care, while the range of scores for good to fair care is from 45-65% [9, 16, 22].

### Patient exit survey and health measures

Finally, we sampled patients who were seen by physicians who completed CPVs. At a randomly selected subset of 100 polyclinics, patients who were seen by one of the study physicians on the day of the survey were sequentially invited to participate, until the target of 5 patients per study physician (a total of 20 exit surveys per facility) was complete.

The patient exit survey contained the same general self-reported health status (GSRH) measure used in the household survey. It employed a five point ordinal scale (translated as “Very good”, “Good”, “Satisfactory”, “Bad” and “Very Bad”) to describe health status. GSRH has been shown to predict objective measures of health, mortality and health care utilization [21, 25]. Additional objective health measures were collected from household survey participants. These included height and weight (and the resulting body-mass index (BMI)), waist circumference, and blood pressure.

We found that those individuals using the facilities (from the exit survey compared to the household survey) were more likely to be women, older, unmarried, Russian speaking, less educated and unemployed. They also lived further from the facilities and reported worse health status, were less likely to smoke, have more chronic conditions, and had three times as many hospitalizations in the past year. From the household survey, we observed heavy drinking rates (17.6% having ≥5 drinks one or more days in the past month) along with a sedentary lifestyle (71.6%). One third to half were overweight, depending on the metric used (See Table 3).

**Table 3:** **Patient characteristics by exit interviews and household survey respondent characteristics**

	Exit interviews	Household interviews
	N = 1932	N = 3430
**SOCIO-DEMOGRAPHIC CHARACTERISTICS**		
**Gender (male) %**	39.3	44.7
**Age**	47.7	41.1
	[17.2]	
**Married (%)**	62	71.5
**Language spoken at home (%):**		
Ukrainian	45.6	48.4
Russian	41	37.9
Both	10.6	11.8
Other	2.7	1.9
**Currently employed (%)**	46.3	63.2
**Education Level (%)**		
Primary	11.7	4.0
High School	52.9	46.2
College	32	42.6
Graduate School	3.5	7.2
**CARE SEEKING BEHAVIORS**		
**Travel time to facility (%)**		
<30 Minutes	61.6	68.0
30 minutes to <1 hour	31.3	24.7
1 to 4 hours	6.9	6.6
>4 hours	0.1	0.8
**Outpatient visits in previous 12 months (mean)**	3.6	N.A.
	[3.5]	
**Outpatient visit in past 30 days (% yes)**	NA	16.6
**Outpatient visits in past 30 days (mean)**	NA	1.5
**HEALTH STATUS INDICATORS**		
**GSRH (%)**		
Very Good	1.4	2.9
Good	22.3	37.7
Satisfactory	52.6	48.2
Bad	22	10.1
Very Bad	1.7	1.0
**Number of Chronic Conditions**	1.4	0.5
	[1.3]	
**Hospitalized in previous 12 months (%)**	34.1	11.0
Average # of hospitalizations overnight care	1.3	1.5
	[1.4]	
Average # of hospitalizations inpatient day care	NA	1.3
**HEALTHY BEHAVIORS AND RISK FACTORS**		
**Excessive waist circumference**	**NA**	**29.5**
(>102 cm Male; >88 cm Female)		
**Excessive alcohol consumption** (5+ drinks for 1+ days in past month)	NA	17.7
**Physical activity**	NA	
Sedentary		71.8
Insufficient		24.0
Active		4.3
**Diet**	NA	0.8
**BMI**	NA	
mean		26.2
< 25		46.9
25-29.9		31.9
>30		21.1
**Smoking status (%)**		
Daily	16	27.1
Occasionally	14.9	4.9
Nonsmoker	69.1	67.9
**Heavy drinker (%)**	NA	17.6
**Activity (% of Sedentary**)	NA	71.6
**Healthy diet**	NA	0.8

All study instruments were translated into Russian and Ukrainian, back-translated to English, and piloted to ensure they were clear and understandable.

### Statistical analyses

Our analyses tested two hypotheses. The first is that the quality of care provided for NCDs (measured by physicians’ CPV® vignette scores) is a function of structural quality measures, physician characteristics, and control variables. We did this because structural inputs, while relatively easy to measure, do not necessarily predict the quality of clinical care or lead to improved outcomes [16]. We estimated CPV® scores as dependent variables, with physician and facility characteristics and control variables for oblast, facility type, and rurality as independent variables.

Quality of care (CPV Score) = ß_0_ + ß_1_ Case complexity + ß_2–8_ Oblast + ß_9_ Setting + ß_10_ Facility Level + ß_11–14_ Facility Infrastructure + ß_15–17_ MD characteristics + ß_18–19_ MD training + ß_20–22_ MD Workload + ϵ.

COPD and CHF were modeled separately to account for the difference in clinical skills required to care for these two conditions. Generalized estimating equation regression models were used, with corrections to account for clustering of physicians within facilities.

The second hypothesis was that improved quality of care for NCDs was linked to improved health outcomes. We estimated models with GSRH as the dependent variable, quality (measured by CPV® vignette scores, averaged for COPD and CHF) as the main independent variable, and patient or household survey respondent characteristics as control variables.

Patient exit survey data were linked to the physician and health facility where each patient was seen. Because it was not possible to ascribe a household respondent to a particular physician, we linked household survey data with the polyclinic(s) serving each household survey EA. Most EAs were served by one polyclinic each, and we used the average of all CPV® vignette scores from that facility. If one polyclinic served two EAs, it was linked to each EA; if two polyclinics served the same EA, we averaged CPV® vignette scores across both facilities.

Two models were separately fit for exit survey patients or household survey respondents.

Exit Survey: Health Status (GSRH) = ß_0_ + ß_1_ CPV score + ß_2–8_ Oblast + ß_9–15_ Oblast*CPV interaction + ß_16_ Facility Level + ß_17_ Setting + ß_18–24_ Patient Characteristics + ß_25_ Diagnostic Index + ß_26–28_ Healthcare utilization + ß_29_ Smoker + ϵ

Household Survey: Health Status (GSRH) = ß_0_ + ß_1_ CPV score + ß_2–8_ Oblast + ß_9–15_ Patient Characteristics + ß_16_ Diagnostic Index + ß_17–18_ Healthcare utilization + ß_19–20_ Objective Health Measures + ß_21–23_ Healthy Behaviors + ϵ

Ordered logistic regression was used for both models, as general self-reported health status (GSRH) was the dependent variable. The household model also included objective (BMI and waist measurement) measures of health to control for pre-existing conditions that might skew the self-reported measures. The distribution of GSRH results was normalized within each oblast. The exit survey model was corrected to account for clustering of respondents by physician.

All statistical modeling was carried out using STATA v11.2 (StataCorp, College Station, Texas, USA). Approval for data collection and analyses were obtained from the Ukraine Ministry of Health. The Oregon State University Institutional Review Board determined that this study does not require review as human subjects research.

## Results

Physicians scored 47.4% on the CPV® vignettes, averaged by complexity and disease type. They had higher scores on CHF (53%) than COPD (42%). For both diseases, Physicians scored higher on simple than complex cases, although this difference was attenuated for CHF. The physician score distribution was approximately normal across disease types and complexity. When we examined scores by domain, physicians did the best at taking a history and ordering appropriate laboratory tests; they struggled the most with treatment and management. For example, for the CHF case, only 45.5% of physicians prescribed an ACE inhibitor and only 27.5% counseled the patient on weight loss. For the COPD case, albuterol was prescribed only 47% of the time and steroids 33% of the time. There were only modest differences between physicians in hospitals versus polyclinics, including diagnostic abilities, although scores were always higher at oblast hospitals (See Table 2).

Regarding our first hypothesis of the effect of facility and physician characteristics on quality, we found that physician-level determinants of higher CPV® vignette scores were age, gender, and relevant continuing medical education (CME)—younger, female physicians and those who had CME in chronic disease or health behaviors in the past year provided higher quality care (Table 4). Specialist physicians performed better on CHF, but not on COPD.

**Table 4:** **GEE Regression analysis of Quality from CPV® scores, by case type**

	COPD		CHF	
	n = 944		n = 944	
	Coef	p-value	Coef	p-value
Constant	43.89	0.00	49.93	0.00
CPV Taken is Complex	**-1.50**	**0.05**	-0.70	0.35
Oblast = Winnitca	**4.33**	**0.03**	**9.98**	**0.00**
Oblast = Dnipropetrovs'k	3.40	0.16	**7.14**	**0.00**
Oblast = Kyiv	-0.79	0.76	4.13	0.09
Oblast = Lugansk	**6.25**	**0.00**	**10.06**	**0.00**
Oblast = Lviv	1.89	0.40	**6.12**	**0.01**
Oblast = Odessa	**4.08**	**0.05**	**9.56**	**0.00**
Oblast = Rivne	0.50	0.84	**5.44**	**0.02**
Urban	-0.05	0.97	1.15	0.26
Hospital	0.60	0.59	0.30	0.80
Equipment index	0.02	0.48	-0.02	0.55
Lab index	-0.01	0.65	-0.01	0.58
Supply index	0.02	0.49	0.03	0.33
Number of Committees	-0.40	0.21	0.17	0.58
Age of MD	**-0.15**	**0.00**	**-0.14**	**0.00**
MD is Male	**-2.22**	**0.03**	**-2.76**	**0.00**
MD Speaks only Russian	-1.31	0.27	**-2.06**	**0.05**
MD is generalist	-2.23	0.18	**-2.80**	**0.05**
With CME at least 3 years age that lasted at least two days	**2.38**	**0.01**	**2.22**	**0.02**
Prop of MD patient age 55 and above	0.04	0.11	0.03	0.18
MD works in other facility	-0.81	0.61	-1.68	0.21
W/ record for at least 75% of patients	0.73	0.45	1.12	0.22

Analysis takes into account clustering by facility.

None of the facility-level structural characteristics—equipment, lab test availability, supplies, or number of committees (a proxy for organizational integration)—had a significant impact on the quality of care. No significant differences in quality were observed between urban-serving and rural-serving facilities, or between hospitals and polyclinics. There was large regional variation in quality when oblasts were compared to Crimea, which had the lowest CPV® vignette scores. Quality did not vary significantly if physicians worked in other facilities, had a higher proportion of elderly patients, or consistently had a medical record available for patient visits.

Regarding our second hypothesis that higher quality is linked to better outcomes, we found that higher CPV® vignette scores were significantly associated with better GSRH among both exit survey patients (Table 5) and the general adult population (Table 6). Regional variation was more often significant in the household survey model than in the exit survey. Patient or respondent age, gender, current employment, and level of wealth had a significant impact on GSRH in both models. The number of chronic conditions and recent hospitalization were also significant in both models. Facility type and travel time to the nearest facility were available only in the exit survey and were significant. In the household survey model, we included several respondent level variables not available from patient exit survey. Having sought medical assistance within the past 30 days was significantly associated with GSRH, but BMI, waist circumference, and being a heavy drinker were not. Smoking and marital status were not significantly associated with GSRH in either model.

**Table 5:** **Impact of quality on self-reported health status using general self-reported health status: exit survey**

	Exit survey	
	N = 1762	
	Odds ratio	p-value
Actual CPV score (Average of COPD & CHF)	**1.03**	**0.04**
Oblast = Winnitca	2.02	0.61
Oblast = Dnipropetrovs'k	2.33	0.29
Oblast = Kyiv	1.66	0.57
Oblast = Lugansk	2.13	0.39
Oblast = Lviv	2.73	0.33
Oblast = Odessa	1.62	0.60
Oblast = Rivne	0.71	0.73
CPV*Oblast = Winnitca	0.97	0.22
CPV*Oblast = Dnipropetrovs'k	**0.95**	**0.00**
CPV*Oblast = Kyiv	0.99	0.48
CPV*Oblast = Lugansk	0.97	0.12
CPV*Oblast = Lviv	0.97	0.12
CPV*Oblast = Odessa	0.99	0.45
CPV*Oblast = Rivne	0.97	0.18
Hospital	**0.63**	**0.01**
Urban	0.87	0.34
Patient's age	**0.97**	**0.00**
Patient is male	**1.53**	**0.00**
Patient is married	1.02	0.86
Patient speaks mainly Russian	1.02	0.85
Employed	**1.27**	**0.04**
Years of schooling	**1.09**	**0.00**
Asset index	**0.53**	**0.00**
Diagnosis index^‡^	**0.66**	**0.00**
2	**0.48**	**0.00**
3	**0.37**	**0.00**
4+	**0.28**	**0.00**
With hospitalization	**0.55**	**0.00**
Travel time = at least one hour	**0.69**	**0.05**
Patient is smoker	0.92	0.47

**Ordered logit** accounting for clustering at the physician level.

Oblast and CPV*Oblast comparator = Crimea; ^‡^Number of chronic diseases, comparator = 0–1 diseases.

**Table 6:** **Impact of quality on self-reported health status using general self-reported health status: household survey**

	Household survey	
	N = 2844	
	Odds ratio	p-value
Average CPV score (average of CPVs at the facility level)	**1.02**	**0.05**
By region:		
Oblast = Winnitca	**1.97**	**0.00**
Oblast = Dnipropetrovs'k	**4.04**	**0.00**
Oblast = Kyiv	**2.65**	**0.00**
Oblast = Lugansk	**1.44**	**0.05**
Oblast = Lviv	1.34	0.12
Oblast = Odessa	0.82	0.24
Oblast = Rivne	**1.49**	**0.02**
Age	**0.96**	**0.00**
Male	**1.45**	**0.00**
Married	0.94	0.44
Speaks mainly Russian	0.99	0.93
Employed	**1.37**	**0.00**
Educ = Primary Level	**0.74**	**0.10**
Lowest wealth quintile	**0.76**	**0.00**
Number of chronic conditions	**0.43**	**0.00**
W/ medical assistance in past 30 days	**0.47**	**0.00**
With hospitalization	**0.46**	**0.00**
BMI	0.99	0.78
Waist risk	0.85	0.12
Smokes	0.90	0.25
Heavy drinker	0.91	0.33
Sufficient physical activity	0.82	0.26

Oblast and CPV*Oblast comparator = Crimea.

## Discussion

Ukraine’s ongoing mortality crisis is driven by the high prevalence and inadequate treatment of NCDs [26, 27]. A growing body of international evidence suggests that improved quality of care can significantly improve the diagnosis and treatment of such diseases, and thereby improve population health outcomes [28]. This study therefore measured the quality of care for two NCDs with a high burden of disease in Ukraine, and addressed two important questions for health policy:

1. How do health care facility and physician characteristics impact quality?
2. Does quality impact the health of clinic patients and/or the general population served by these clinics?

This study used a previously validated method—Clinical Performance and Value (CPV®) vignettes—to measure the quality of care for COPD and CHF. Quality was measured at a large number of primary care sites and hospital outpatient clinics across Ukraine, sampled based upon a prior household survey. At the study facilities, additional surveys measured facility and physician characteristics that might impact quality. Patients seen by physicians completing CPV® vignettes were asked to complete exit surveys, and the household survey provided health data about the general adult population.

Overall, the quality of care for COPD and CHF in Ukraine is substandard, with average scores of 47.4% being below the 50-60% range typically observed in other countries [29] or the average scores of 60.2-62.6% reported for communicable and perinatal conditions in a five-country study [16]. Scores in Ukraine are comparable to the 47.7-48.4% range seen in Macedonia, another Eastern European setting [7], but well below those of 65% for COPD and 70% for vascular disease observed in the United States [14]. Other investigators have also found COPD care in Ukraine to be of poor quality [30]. Consistent with CPV® results from other countries, we observed wide variation across providers within Ukraine, with many scores of 65 and above, particularly at oblast hospitals.

At the facility level, structural characteristics did not predict quality scores, a finding consistent with other studies [9]. There were also not significant differences in quality between urban- and rural-serving facilities, or between hospitals and polyclinics. There was, however, significant regional variation in quality across oblasts.

At the physician level, recent continuing medical education had a positive impact on the quality of care provided. Similar to results from other countries, younger and female physicians had higher scores, but other physician characteristics were associated with quality levels. Interestingly, working at another facility did not impact quality.

At the patient and population level, higher quality was associated with better health status, controlling for age, education, employment, and wealth. Specifically, higher physician CPV® vignettes cores were associated with better GSRH among clinic patients, and higher scores at facilities were positively associated with GSRH among adults in neighborhoods served by those facilities. As expected, younger age, less chronic illness, and lower prior utilization of health care were associated with better health status. Somewhat surprisingly, health behaviors or objective health measures had a very limited impact on health status after controlling for other factors.

These findings have several implications for health policy in Ukraine and other LMICs. Most important, higher quality of care can mitigate some of the more intransigent socioeconomic determinants of health, making quality improvement an important policy goal to overcome inequities in both service provision and disease burden. The low treatment domain scores we observed, coupled with the widely demonstrated impact of inexpensive secondary prevention on improving health outcomes for NCDs such as CAD, CHF, and COPD [31], suggest that improved quality of care is a practical objective for health policy.

Specifically, health system investments and payment reforms can emphasize improvements in clinical practice. Expansion of primary care increases the pool of new physicians trained in treating NCDs [32], and CME can improve such skills among the existing physician workforce. Payment mechanisms can also encourage the provision of NCD care that meets international standards. For example, when quality is measured and the results fed back to physicians, incentives and interventions have been shown to lead to rapid improvements in clinical practice and patient outcomes [22, 33].

The non-significant effect of structural measures indicates that policies directly targeting clinical practice can operate independent of investments in new or renovated facilities. This does not suggest foregoing investments in equipment and supplies, but instead highlights that material inputs are not necessarily dominant over actual clinical practice.

Finally, our results point out the need to target policies on a regional basis. The regional variation observed in Ukraine was greater than the rural–urban quality differential seen in other countries. Therefore, even though nearly all physicians could benefit from, for example, additional CME in COPD and CHF, analyses such as those presented here can be used to better inform targeting additional resources to regions with lower CPV® scores.

There are limitations to this study. Its main health outcome is self-reported health status; ideally this measure is also accompanied by objective health measures, which for budgetary reasons was not possible for exit survey patients. Exit survey patients were not initially screened for COPD or CHF, and may not be fully representative of Ukraine residents with those conditions. Future studies would gain power by stratifying patients by the diseases of interest. For the household survey, we are not able to confirm that respondents attended the sampled facilities, even though households were linked to facilities by enumeration area. If physicians who refused to participate would have had lower CPV® scores than participating physicians, the actual quality of care provided may be even lower than estimated in this study. Finally, debate continues on whether vignettes measure knowledge or practice. The CPV® vignettes are explicitly structured to address this concern, and validation studies have been designed and implemented in a number of settings showing that CPV® vignettes do measure practice [14]. Perhaps most importantly, this study provides only a snapshot at one point in time. Policy efforts would greatly benefit from evaluating the impact of different approaches on the quality of care and population health.

## Conclusions

As LMICs face a growing burden of non-communicable diseases, addressing the quality of care will be an important policy priority. This study’s evidence of linkage between quality and health status suggests that policy makers should strongly consider approaches that directly aim to improve the quality of care health services and thereby enhance population health.
